# A Support Tool for Emergency Management in Smart Campuses: Reference Architecture and Enhanced Web User Interfaces †

**DOI:** 10.3390/s24185887

**Published:** 2024-09-11

**Authors:** Giovanni Delnevo, Vittorio Ghini, Enrico Fiumana, Silvia Mirri

**Affiliations:** Department of Computer Science and Engineering, University of Bologna, 40126 Bologna, Italy; vittorio.ghini@unibo.it (V.G.); enrico.fiumana@unibo.it (E.F.); silvia.mirri@unibo.it (S.M.)

**Keywords:** smart campus, emergency management, people-counting system, Internet of Things

## Abstract

In the context of smart campuses, effective emergency management is crucial for ensuring the safety and well-being of students, staff, and visitors. This paper presents a comprehensive support tool designed to enhance emergency management on smart campuses, integrating a low-cost people-counting system based on cameras and Raspberry Pi devices. It introduces a newly designed architecture and user interfaces that enhance the functionality and user experience of a smart campus disaster management system. Finally, a usability evaluation has been carried out to validate the brand-new user interfaces devoted to emergency management.

## 1. Introduction

Every disaster demands effective management to safeguard people and property [1,2]. Thankfully, the rise of Internet of Things (IoT) technology brings new possibilities for handling emergencies across various fields, including universities equipped with smart technology systems [3,4,5].

Smart campuses are often seen as scaled-down versions of smart cities, functioning as living laboratories for the research, development, and adoption of smart technologies [6]. Hence, taking advantage of integrated IoT techonologies, smart campuses can efficiently respond to and mitigate the impact of emergencies [7]. In essence, this network of sensors can be used for early warning systems that constantly monitor environmental factors and seismic activity [8]. By identifying potential disasters early, automated alerts and notifications can be sent to the right people. This proactive approach strengthens the smart campus’s ability to prepare for and respond to emergencies.

The IoT takes disaster management to a new level with its data analysis and prediction capabilities [9]. Inter-organizational communication and coordination have long been of great interest in emergency management [10], and the IoT can serve as a facilitator [11]. It can facilitate real-time communication between individuals, emergency responders, and campus authorities, ensuring the rapid dissemination of critical information during emergencies, including safety exits and emergency procedures [12].

Furthermore, IoT devices can play a crucial role in enhancing campus safety by enabling real-time tracking and location data of individuals. This capability is particularly valuable during emergencies, as it allows authorities to quickly identify the whereabouts of students, faculty, and staff, facilitating efficient rescue operations [13,14].

Such a flood of information from IoT devices can be sifted through to uncover patterns, trends, and potential threats [15]. Machine learning can be used to create models that predict the chance and intensity of disasters, allowing for proactive steps to be taken [16,17]. Data analysis is also valuable after a disaster, helping to develop plans for future prevention and recovery [18,19].

It is important to consider that IoT devices can be connected to existing systems like fire alarms, security gates, and cameras to make the whole smart campus better more prepared for emergencies [20]. This allows everything to work together in real time, so the system can automatically react to situations. For instance, during a fire, IoT devices could turn on sprinklers, call firefighters, and show people the quickest way out [21]. By combining these systems, the campus creates a powerful network that makes disaster management much more effective.

Therefore, using IoT for disaster management in a smart campus is a game-changer for safety, response, and recovery [22]. Imagine a network of devices, sensors, and data analysis all working together to spot trouble early, share information quickly, make smart decisions, and keep the campus prepared [23]. However, there are still hurdles to jump, like data privacy, security, and making sure all the systems work together [24]. With careful planning and ongoing improvements, though, IoT disaster management has the potential to make smart campuses much safer and better able to handle emergencies.

In a previous work [25], we explored the use of a people-counting system within a smart campus to enhance emergency management processes. The aim of this research is to leverage an existing low-cost people-counting system as a foundational component to develop a support tool specifically designed for emergency management within smart campuses. The rationale behind this approach is twofold. On the one hand, by building on an existing people-counting system, we are able to optimize resources and reduce costs, making the emergency management support tool more accessible and feasible for implementation in various smart campus environments. On the other hand, the integration of the people-counting system into our support tool provides critical real-time data that can be used to monitor and manage situations more effectively during emergencies. The discussion encompassed its potential contributions to handling various emergencies, including fires, earthquakes, and floods. Additionally, the paper delved into its role in managing pandemics, exemplified by the COVID-19 outbreak. Lastly, critical issues pertaining to the existing people-counting system were presented, highlighting the need for addressing or mitigating these challenges for the system’s effective implementation in emergency management.

In this paper, starting from the scenarios presented in [25], we provide a technical contribution regarding the system architecture and the User Interface (UI). The proposed system architecture is tailored specifically for disaster management within a smart campus environment and has the main aim of increasing the reliability of communication, maintaining the low-cost aspect of the system. With regard to the UIs, we propose a brand-new UI, tailored to support emergency management. These interfaces have been designed and evaluated with a focus on usability, and have been tested through a study conducted to validate their effectiveness.

The rest of the paper goes as follows. Section 2 discusses some related works focused on emergency management using IoT from a general perspective and in the smart city and in smart campuses contexts. Then, Section 3 details the previous architecture of the people-counting system, together with the requirements that drove this study. Then, Section 4 illustrates the proposed architecture and the new UIs designed as an emergency management support tool. Finally, Section 5 wraps up the paper, offering final remarks and outlining potential directions for future work.

## 2. Background and Related Work

This Section presents some works related to (i) the use of IoT technologies for emergency management, (ii) emergency management in the context of smart cities, and (iii) emergency management in the realm of smart buildings and smart campuses.

### 2.1. IoT for Emergency Management

The growing interest in leveraging IoT technologies for emergency management has gained significant momentum in recent years, as organizations and institutions recognize the potential of connected devices to enhance safety and response capabilities. The ability of IoT systems to provide real-time data, automate alerts, and facilitate communication during crises has positioned them as a crucial component in modern emergency management strategies. This burgeoning interest is also demonstrated by the First International Workshop on Internet of Things for Emergency Management (2020), whose results have been presented in [26]. The discussion session among workshop participants and paper presenters highlighted key challenges, including (i) addressing issues related to the installation and coverage of IoT resources; (ii) understanding the impact of environmental context on detection; and (iii) meeting the real-time requirements. Liu and Wang directed their attention to urban emergencies, specifically emphasizing traffic emergency responses in their work [27]. They presented a system designed to bolster urban emergency management by gathering data from various sources to facilitate emergency respondents’ coordination. Cheikhrouhou et al. introduced a cloud-based system that integrates wireless sensor networks (WSNs) with 3D virtual environments for natural disaster management [28]. The system collects real-time data from WSNs to create a realistic 3D environment, facilitating the training of rescue teams in various scenarios. It employs an efficient cloud architecture, combining 3D data streaming and sensor data collection. Notably, the system utilizes an enhanced Routing Protocol for Low-Power and Lossy Networks (RLP) for data transfer and a dynamic game engine for near-real-time 3D rendering. An Extensible Markup Language (XML) atomic action concept allows continuous scene modifications without interrupting the engine. Additionally, the paper proposes a multi-objective multiple travelling salesman problem (AHP-MTSP) algorithm for generating efficient rescue plans. Results show that immediate feedback from the 3D environment aids in preparing effective rescue plans with optimal resource allocation. Jia and Wu [29] implemented a neural-network-based system for emergency detection with the aim of minimizing human-induced inefficiencies in emergency systems. An example of this smart government system has been tested in real settings to prove its effectiveness.

Contrastingly, Yang et al. [30] tackled emergency management from the opposite side, developing a user-centered smart emergency response. Such a system continuously monitors the health status of users and triggers warnings when emergencies are detected.

### 2.2. Emergency Management in Smart Cities

Emergency management in smart cities provides valuable insights and foundational strategies that can be adapted and applied to the specific context of smart campuses. The integration of advanced technologies into emergency management systems is a critical aspect of modern smart cities. Elvas et al. [31] present an integrated resilience system for smart cities that enhances disaster recovery by linking interconnected critical infrastructures. Using a data-driven approach and artificial intelligence, the system aims to reduce cascading failures and rapidly restore infrastructure performance. It provides a decision support system that improves disaster preparedness, response, and recovery by leveraging the interconnections between critical infrastructures. The approach is illustrated through a case study of Lisbon, Portugal.

Building on this, the concept of smart cities often focuses on enhancing community life through continuous collaboration among citizens, services, and organizations. In this context, emergency management becomes crucial. Romano et al. [32] discuss the redesign of an Emergency Notification (EN) application within smart cities, focusing on enhancing community collaboration and emergency management. The redesign incorporates gamification and Self-Determination Theory (SDT) principles to improve user experience and encourage civic participation. By connecting citizens, services, and organizations, the application aims to reduce emergency risks and response times. The effectiveness of the new design was assessed through an exploratory focus group with citizens and practitioners.

Moreover, De Nicola et al. [33] proposed a framework that supports the creative design of emergency management scenarios by automatically generating and organizing conceptual models, called “mini-stories”. These mini-stories, derived using semantics-based techniques, help in modeling and simulating emergency situations, particularly in smart cities. The framework integrates structural, domain, and contextual knowledge to assist in creating detailed scenarios, addressing the complexity of smart city ecosystems. A software application was developed to facilitate this process, and the approach was validated through experiments with city planners.

Finally, the development of smart city digital twins is another innovative approach to disaster management. Ford and Wolf [34] explored the use of digital twins in smart cities (SCDT) for enhancing community disaster management. They proposed and tested a conceptual model of an SCDT tailored for disaster scenarios and identified key challenges and mitigation strategies related to SCDT development. The study emphasizes the importance of focusing on information loops rather than individual components. Key contributions include a framework for SCDT in disaster management, a detailed conceptual model, and a discussion of development and deployment issues.

### 2.3. Emergency Management in Smart Campuses

Narrowing the context further, numerous studies have delved into the role of Information and Communication Technology (ICT) systems in smart campus emergency management. Zhang et al. [35] carried out a systematic review to reveal prevailing research patterns in smart campuses. The review was carried out along two main dimension of analysis. The former one examined the enabling technologies for smart campuses. This analysis aimed to reveal which tools and technological innovations are essential for creating a campus environment that fully leverages the potential of the Internet of Things (IoT), data analytics, and advanced communication systems. The latter consists of application domains within the smart campus concept. This examination looks at how smart technologies can be applied across various sectors, from enhancing security and energy management to creating more engaging and personalized learning experiences. Additionally, they presented a case study that adheres to the human-centred principle of smart campus development, evaluating its consistency and alignment with current research trends. A different survey was carried out by Munawar et al. [36]. The authors discussed and evaluated tools and technologies to analyze big data efficiently, focusing on field-specific applications: smart real estate and disaster management. The proposed framework presents several scenarios where big data coming from heterogeneous sources can be used to tackle emergencies.

Recent research has explored the potential of social media to revolutionize emergency management strategies [37,38]. Bukar et al. [37] investigated the role of social media for emergency management in smart campuses. Building upon existing research assessing crisis communication theories and models, the authors discuss contemporary opportunities and challenges encountered by universities and educational institutions during crises. The authors emphasize the critical role of social media in crisis communication plans and advocate for future research to explore effective strategies for integrating social media into emergency response efforts. Ramirez et al. [38] introduced a study centered on treating users as valuable assets by leveraging their social media engagement. The authors presented a system designed to detect emergency events at the National Polytechnic Institute Zacatenco. This system classifies Twitter messages from users near the area of interest using three machine learning models (Bayes Multinomial, Support Vector Machines, and k-Nearest Neighbors) into four classes (mobility, fire, health, and none).

Moving from social media to IoT, Hannan et al. [39] proposed an NDN-based IoT-DMS architecture specifically designed for fire disasters, named NDN-DISCA. Within NDN-DISCA, producers in the NDN architecture actively disseminate emergency content to nearby consumers. To facilitate push support, a Beacon Alert Message (BAM) is generated using fixed sequence numbers. The proposed NDN-DISCA architecture is simulated using ndnSIM, focusing on a disaster scenario within an IoT-based smart campus (SC). The simulation results indicate that NDN-DISCA demonstrates minimal delay and improved throughput compared to both legacy NDN and existing PUSH schemes. This underscores the potential efficacy of the NDN architecture in enhancing disaster management within the IoT framework.

In conclusion, Narendrakumar and Pillai [40] detailed the adoption of smart city technologies and services within a university campus. The application “Campus Info” is the central component of the emergency management system. It analyzes data from various monitoring systems to deliver timely information and support. By providing these crucial details promptly, it helps users to stay informed and, in case of an emergency, allows them to request assistance quickly by connecting directly to ambulance services, campus security, or designated university contacts.

## 3. Materials and Methods

This Section details the previous architecture of the people-counting system installed on the campus and the requirements for emergency management that have driven the design of the architecture and the new User Interfaces.

### 3.1. Previous Architecture of the People-Counting System

The case study for our research focuses on the campus of Cesena, part of the University of Bologna. The architecture of the people-counting system was proposed and described in depth in [41]. Here, we summarize the main components of such a system, their organization, and how they interact with each other. In [41], the architecture of the people-counting system is been thoroughly detailed, together with the main UIs. In this section, we provide an overview of the system’s key components, their organization, and their interactions.

The system is designed with three layers, each serving a distinct function. The initial layer is the data acquisition layer, utilizing Intel RealSense D415 Depth cameras connected to a Raspberry Pi 4 Model B via USB. These cameras capture high-resolution images at 1280 × 720 pixels every five minutes. This interval is set to optimize storage management, though it can be modified to suit specific operational requirements. This layer is responsible for collecting raw visual data from the environment, which forms the foundation for subsequent processing stages. The second layer is the prediction layer, which processes the images collected by the cameras. This layer employs a custom model based on YOLOv3 (You Only Look Once version 3), an advanced object detection algorithm. The model analyzes the images to detect and count the number of individuals present. It achieves this by segmenting each image into various regions, applying bounding boxes around detected figures, and assigning confidence intervals to each detection. The YOLOv3 model has been fine-tuned through transfer learning to enhance its accuracy in the specific context of this application. The processed results, including the number of detected individuals and corresponding timestamps, are temporarily stored in a CSV file for later retrieval. The final layer is the API layer, which is built around an HTTP server. This server handles requests for data and retrieves the information stored in the CSV file. When a request is made, the server accesses the CSV file to extract the relevant data and sends it back to the requester. This layer acts as the interface through which users can access the processed information from the prediction layer, facilitating easy and efficient data retrieval.

The three-layer architecture follows a fat client–thin server model, offering advantages such as increased scalability, semi-offline functionality, enhanced availability, and compliance with privacy regulations.

The current User Interface is built as a web application, primarily devoted to visualizing information about lessons and students who are attending them for each course of study that takes place on the Cesena Campus.

### 3.2. Requirements for Emergency Management

In [25], several scenarios were discussed in which the people-counting system could be effectively employed as a supporting tool for the management of emergencies, including a pandemic, and possible critical issues were also highlighted. In this Subsection, we list the requirements to meet the needs that can arise in emergency scenarios. Such requirements drove the design of the improved system architecture, together with a brand-new User Interface. The following were considered:

- Alert Notification System: A notification system to alert users when a given event takes place. Such a system not only allows users to be warned about emergencies, but also allows the dissemination of general information about campus life, such as seminars and workshops.
- Emergency Mode: Specific users who deal with campus security can activate a specific mode during an emergency. It makes available specific functionalities mainly devoted to emergency management.
- Real-time Data Visualization for Occupancy: A specific data visualization that shows the campus map together with information about the occupancy of rooms and laboratories that is updated continuously. It is the core functionality for the management of emergencies. In fact, it can act as a tool for evacuation assistance and planning, providing real-time information on the number of people present in each classroom. In this way, emergency responders can promptly evaluate classrooms requiring urgent evacuation, enabling them to prioritize their actions according to the occupancy level of each room. This facilitates the effective distribution of resources, directing rescue endeavours towards areas housing a larger number of individuals.
- Post-Emergency Analysis: A detailed report about a specific emergency can be produced. It contains information about occupancy patterns, evacuation times, and response effectiveness, with the aim of providing advanced analytics to improve emergency management strategies and policies.

Moreover, the critical issues that can arise during an emergency and that we want to mitigate are the following:

- System Disruption. The current setup relies on the campus’s existing infrastructure, including its power supply and network connectivity. The system operates on the electrical grid, and the cameras send occupancy data through the university’s Wi-Fi. However, in the case of emergencies, both the infrastructure and the physical components, such as cameras and sensors, could be compromised. This could render the system temporarily or permanently inoperative, impacting its ability to accurately count and monitor individuals. To mitigate such risks, redundancy strategies could be introduced, such as incorporating backup batteries for the Raspberry Pi that manages the cameras or employing an alternative communication system like LoRa. These solutions would help to maintain system functionality and report any issues with power or connectivity.
- Error Reporting System. At present, the system lacks an “alarm” feature to alert users if any node (such as a classroom or lab camera) fails to transmit occupancy data. Enhancements could be made to enable the system to notify administrative staff when a classroom stops sending updates. This would allow personnel to prioritize monitoring efforts on rooms that have not provided recent information.

## 4. Our Prototype

This Section details the proposed prototype, describing the improved system architecture, the UIs designed for emergency management, and a usability evaluation of them.

### 4.1. System Architecture

**Figure 1:**
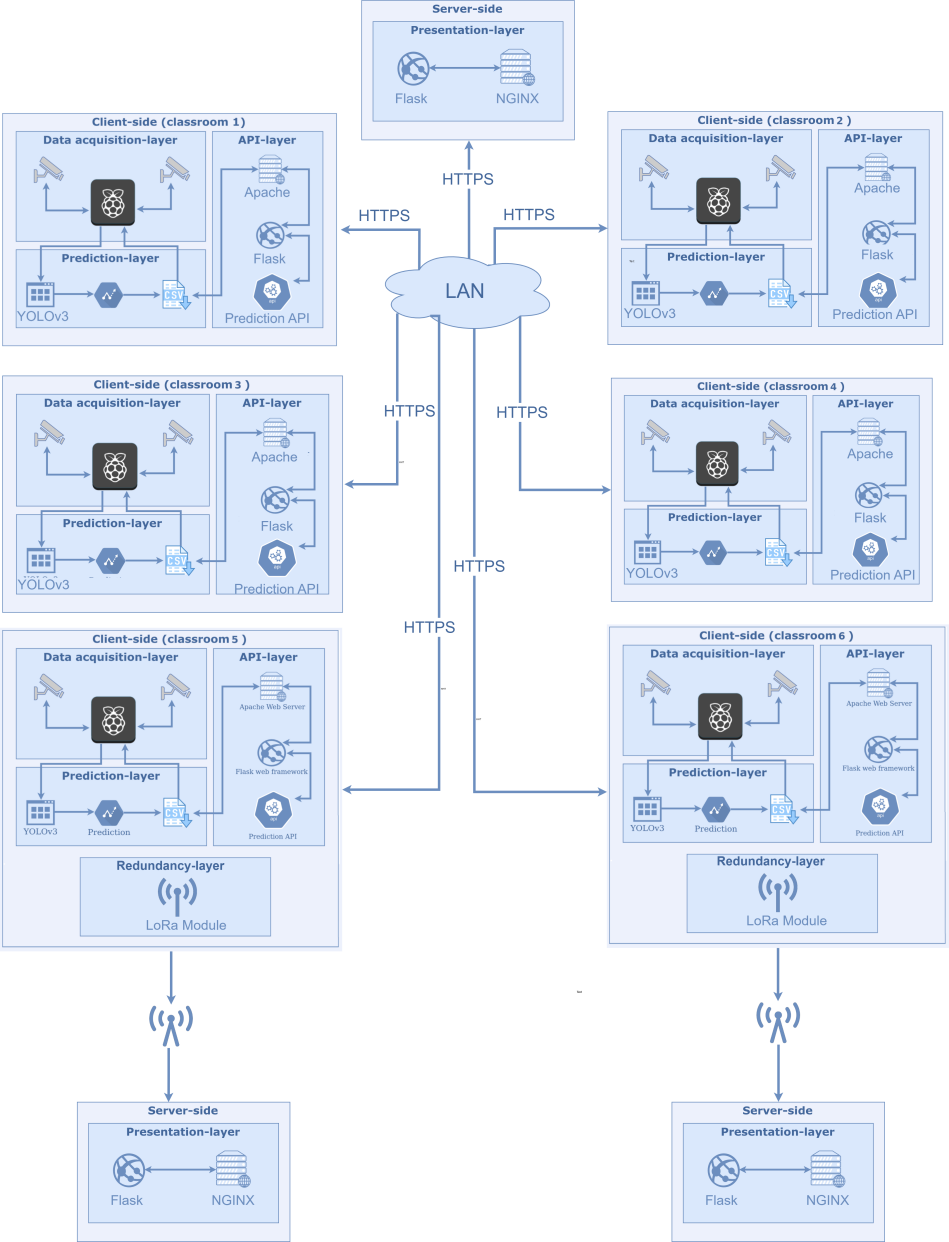
Improved system architecture for emergency management.

The improved system architecture for emergency management is reported in Figure 1. It is always based on a fat client–thin server architecture where the computation load weighs on clients. Each client is equipped with an additional battery to ensure the client’s work even in case of emergency. The system differs from the previous one just due to some adjustments. There are some clients (in the Figure, the ones in classrooms #5 and #6) that have a further layer, the Redundancy one. Such clients were strategically chosen since they are at the opposite end of the campus. The Emergency Data Transfer feature was implemented, which leverages LoRa (Long Range) technology to ensure critical data are preserved and transferred during network failures or other emergency scenarios. LoRa is a low-power, long-range wireless communication technology that provides robust and reliable data transmission over distances significantly greater than are possible with traditional Wi-Fi or Bluetooth solutions. This makes it an ideal choice for ensuring data continuity in a smart campus environment.

Each of these two clients is equipped with a LoRa module. This module enables the Raspberry Pi to communicate with external servers over long distances without relying on the campus’s primary network infrastructure. If a disruption, a failure, or an emergency is detected, the module activates and the Raspberry Pi begins transmitting essential data packets to predefined external servers using the LoRa network.

Such clients, during emergencies, collect data from the other clients of the LAN. Then, such data are transferred via LoRa to maintain the integrity of the people-counting system. The data fit within the limited bandwidth of LoRa communications since they consist of just a list of integers, ensuring efficient and timely delivery. The LoRa network operates on a duty cycle, meaning that data are sent at regular intervals or triggered by specific events, such as a network failure. This ensures that even during extended outages, the most recent and relevant data are transmitted.

The integration of LoRa for the transfer of emergency data offers several advantages. Firstly, it provides a fail-safe mechanism that enhances the reliability and resilience of the people-counting system. In the event of a network failure, critical data are not lost but rather securely transmitted to external servers, where they can be retrieved and analyzed later. This is particularly important for maintaining continuous monitoring and ensuring data-driven decision-making, even in adverse conditions. Moreover, LoRa’s low power consumption is ideal for emergency scenarios where power resources may be limited. The technology’s ability to operate over long distances also ensures that data can be transmitted even if the primary network is compromised. This extended range is particularly useful in large campus environments where network issues might be localized but still affect critical system operations.

The server responsible for receiving data via LoRa plays a crucial role in ensuring the integrity and availability of critical information during network failures or emergencies. This server is specifically designed to handle the unique requirements of LoRa communication, including the low bandwidth and sporadic nature of data transmission. The LoRa data reception server is located in another facility also belonging to the University of Bologna, located two kilometers from the campus, with reliable power and network infrastructure to ensure its continuous operation even when the primary campus network is compromised.

To manage the incoming data, the server runs specialized software capable of handling the intermittent and low-bandwidth nature of LoRa communications. This software is designed to parse and store the data efficiently, ensuring that each data packet is correctly timestamped and associated with the originating client. Once the data packets have been received, the server processes and stores them. Such data are accessible using a web interface that is similar to the one available on the main server of the campus.

Even if this backup communication channel is designed to enhance the resilience of the support tool during emergencies, it is important to recognize the inherent trade-offs in this design. The system’s architecture is carefully balanced to maintain a low-cost solution while providing practical support for emergency management. In this context, LoRa serves as a targeted solution rather than a comprehensive network replacement. The architecture is not designed to ensure that all nodes maintain communication with LoRa-connected nodes in every possible scenario. Instead, it focuses on providing critical connectivity where it is most needed, while accepting certain limitations to preserve the system’s affordability and practicality.

In the event of a severe disaster that results in widespread network failure, including the potential isolation of LoRa-connected nodes from the rest of the system, our design acknowledges these limitations as part of a deliberate strategy. Ensuring full communication coverage in all extreme scenarios would require additional infrastructure and more sophisticated technologies, significantly increasing the system’s complexity and cost. This would fundamentally alter the nature of our system, moving it away from its intended purpose as a cost-effective and accessible support tool for emergency management.

Finally, we want to remark that the focus of this work is to develop a support tool for emergency management that operates with minimal and well-defined requirements. Specifically, the tool is designed to gather and utilize occupancy data, detailing the number of people present in various classrooms and laboratories within a smart campus. While our case study employs cameras and Raspberry Pi devices to collect this information, the system is intentionally designed to be flexible. The web UI we have developed, which will be detailed in the following subsection, relies solely on an API to retrieve occupancy data, making it adaptable to a variety of sensor technologies. The decision to limit the scope of required sensors was driven by practical considerations. Although integrating additional IoT devices, such as fire detection sensors, could enhance the system’s functionality, we recognized that many buildings, including those on the campus of our case study, lack the necessary APIs or interfaces to retrieve data from these existing emergency sensors. This limitation could significantly restrict the applicability of a system reliant on such integrations. By focusing on a solution that can operate independently of these additional sensors, we ensure that our tool remains versatile and can be implemented in a wide range of environments. This approach allows the system to be deployed in buildings that may not have advanced IoT infrastructure while still providing essential support for emergency management.

### 4.2. Web User Interface

**Figure 2:**
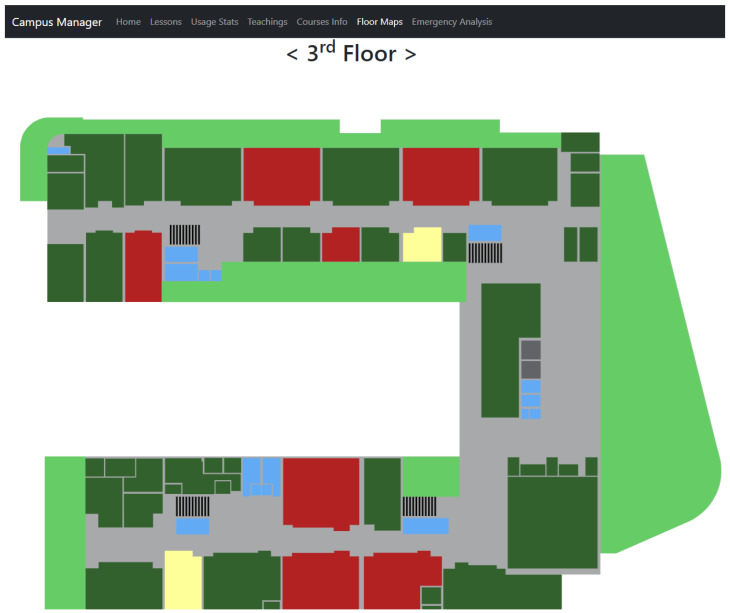
Floor Map user interface.

In addition to the UI already presented in [41], other UIs specifically designed to manage emergencies have been developed: Floor Map and Emergency Analysis. Figure 2 depicts the Floor Map UI.

On the top of the page, there is the floor selection panel which is an essential component designed to offer quick and seamless navigation between different floors of the building. It provides a user-friendly interface for selecting and viewing floor-specific maps. A clear, prominent indicator highlights the currently selected floor, ensuring that users are always aware of the context in which they are operating.

This panel is designed with efficiency in mind, enabling emergency responders to move swiftly between floors without losing crucial time. For buildings with many floors, the panel may include a scrollable list to accommodate all levels without cluttering the interface. The design prioritizes accessibility and ease of use, incorporating features like keyboard navigation and screen reader compatibility to ensure it can be used effectively in high-stress situations. Additionally, the panel might include visual cues, such as colour-coding or icons, to quickly convey the evacuation status of each floor, helping users to identify areas that require immediate attention at a glance.

The floor map display is the central feature, occupying the main area of the interface. This display provides a detailed, scaled map of the selected floor, highlighting the layout of rooms, laboratories, corridors, and other essential areas. Each room and laboratory is clearly outlined, ensuring that users can quickly identify specific locations. The map is designed to be interactive, allowing users to zoom in and out and pan across the floor plan for a closer examination of particular areas.

To facilitate real-time monitoring, it uses a dynamic colour-coding system to indicate the evacuation status of each room and laboratory. Rooms that have been fully evacuated are shaded in green, signalling to emergency personnel that these areas are clear. Rooms that still contain people are highlighted in red, drawing immediate attention to areas where assistance is needed. Additionally, rooms where an evacuation is in progress or incomplete are marked in yellow, indicating that these areas require follow-up. This colour-coded approach provides an at-a-glance overview of the floor’s status, enabling quick decision-making and prioritization of effort.

The floor map display also incorporates interactive features that enhance usability and provide detailed information regarding demand. When users click on a specific room or laboratory, a pop-up window or side panel appears, displaying comprehensive information about the room, such as the number of occupants remaining, any special conditions (e.g., the presence of hazardous materials), and notes from emergency personnel. These interactive elements ensure that users have access to all necessary information without cluttering the main display, maintaining a clean and focused interface. Furthermore, the map is equipped with real-time updates, automatically refreshing to reflect the latest status changes and to ensure that emergency responders have the most current information at their fingertips.

The other UI, Emergency Analysis, is designed to provide comprehensive statistical insights and analytical data related to emergencies. This page serves as a resource for post-event analysis, performance review, and strategic planning, offering detailed numerical data and visualizations that help users understand the effectiveness of emergency responses and identify areas for improvement. It contains several components, listed below from top to bottom.

**Figure 3:**
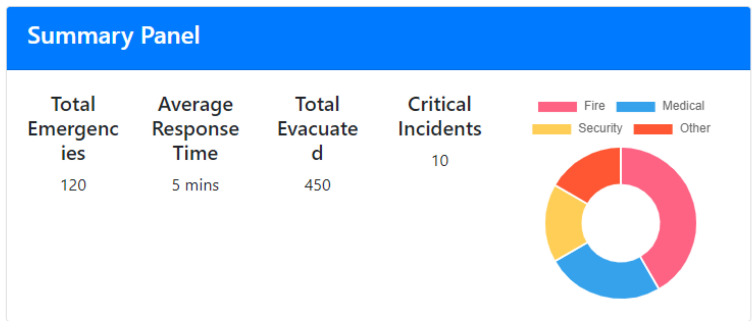
Emergency Analysis—summary panel.

The Summary Panel, depicted in Figure 3, is the top section of the Emergency Statistics page, and is designed to provide users with a high-level overview of key emergency statistics at a glance. This panel is the first point of interaction for users, offering a quick and comprehensive snapshot of recent emergency events and their critical metrics. At the core of the Summary Panel are several key metrics that capture the essence of emergency performance. These metrics include the Total Emergencies recorded over a specified period, giving users an understanding of the frequency of incidents. The Average Response Time metric highlights the efficiency of the emergency response teams, showcasing how quickly they can react to emergencies. The Total Evacuated metric provides an aggregate count of individuals safely evacuated, reflecting the effectiveness of the evacuation protocols. Additionally, the Critical Incidents metric tallies the number of high-severity incidents, indicating areas that may require more focus or resources. The Summary Panel also incorporates a doughnut chart that displays the types of emergencies.

**Figure 4:**
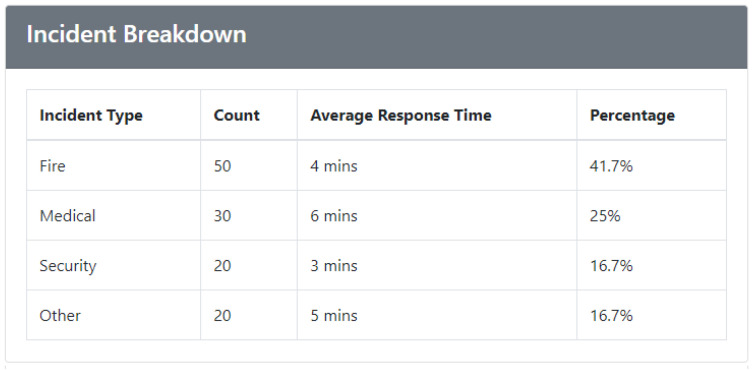
Emergency Analysis—incident breakdown.

The next component of the page is the Incident Breakdown, shown in Figure 4. It offers a detailed and categorized analysis of various types of emergencies that have occurred. This section is critical for users who need to understand the nature and distribution of incidents within the building or campus. By organizing incidents into specific categories such as fire, medical emergencies, security breaches, and others, this section provides a granular view of the different types of emergencies, allowing users to identify patterns and trends. Each category in the Incident Breakdown is accompanied by a comprehensive set of statistics presented in a tabular format. The statistics table includes counts, averages, and percentages for each incident type, providing detailed insights into the frequency and impact of different emergencies. For instance, users can see the total number of fire incidents, the average response time for medical emergencies, and the percentage of security breaches relative to all incidents. This level of detail helps emergency managers and decision-makers pinpoint which types of incidents are most common and where response efforts may need to be focused or improved.

**Figure 5:**
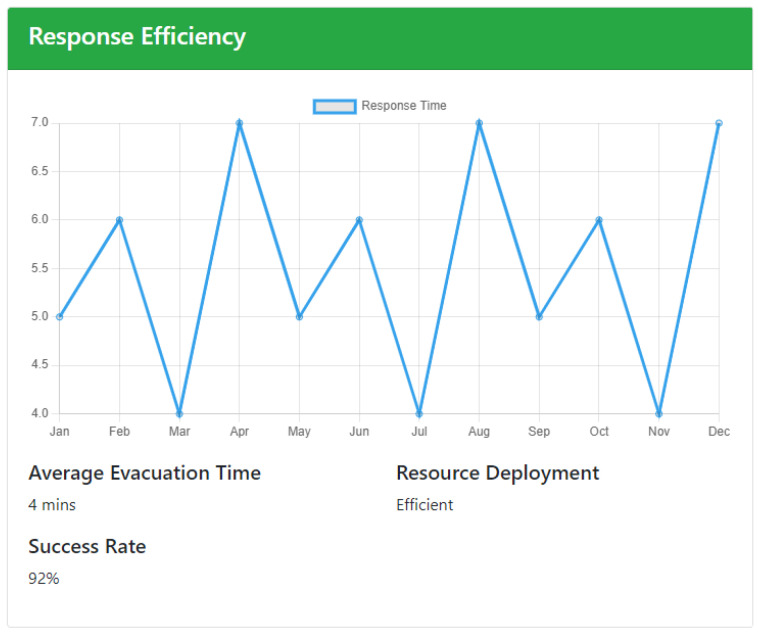
Emergency Analysis—response efficiency.

The next component on the page is Response Efficiency, whose UI is depicted in Figure 5. It lists some metrics to evaluate the speed, efficiency, and effectiveness of the emergency response process, offering insights that are crucial for improving future performance and ensuring the safety of occupants. It is designed to help users understand how quickly and effectively their teams are responding to emergencies, identifying strengths and areas for improvement. It is composed of two main parts. The first one visualizes a line chart that shows response times across different incidents. Users can quickly identify patterns, such as the most common response times or outliers where the response was unusually slow. By analyzing these distributions, emergency managers can assess whether their teams are meeting established benchmarks and identify any factors that may be contributing to delays. The second one reports some metrics, instead. The Average Evacuation Time provides the overall average time taken to evacuate people from affected areas, offering insights into the effectiveness of current evacuation plans and protocols. Additionally, the section includes data on Resource Deployment, such as the number and types of resources (e.g., personnel, medical supplies, equipment) utilized during emergencies. This helps users understand how resources are being allocated and whether they are sufficient to meet the demands of different incidents. Lastly, the Success Rate metric, which indicates the percentage of successful evacuations versus unsuccessful or partial evacuations, provides a clear measure of overall effectiveness. Together, these metrics form a comprehensive picture of response efficiency, enabling data-driven decisions to enhance emergency preparedness and response strategies.

**Figure 6:**
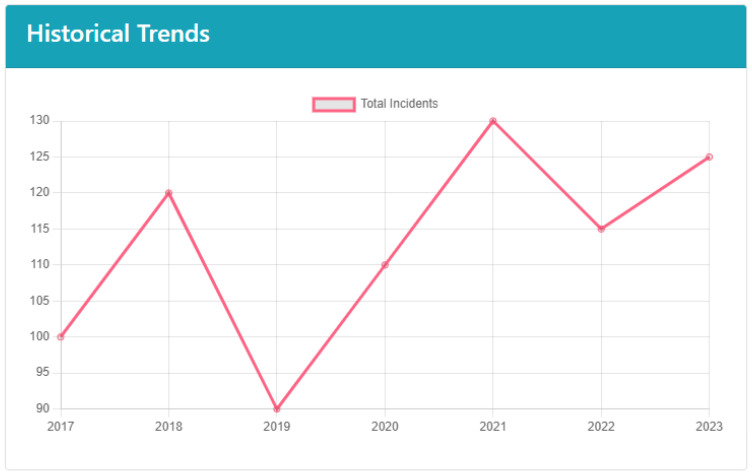
Emergency Analysis—historical trends.

Another component is the Historical Trends section, situated at the bottom of the Emergency Statistics page, which provides a longitudinal view of emergency data, enabling users to analyze how various metrics have evolved over time, as shown in Figure 6. This section is essential for identifying patterns, assessing the effectiveness of past interventions, and making informed decisions to improve future emergency response strategies. By offering a temporal perspective, the Historical Trends section helps users understand the broader context of their emergency management efforts. The Historical Trends, a line chart visualizing the total incidents per year, represent the main content.

**Figure 7:**
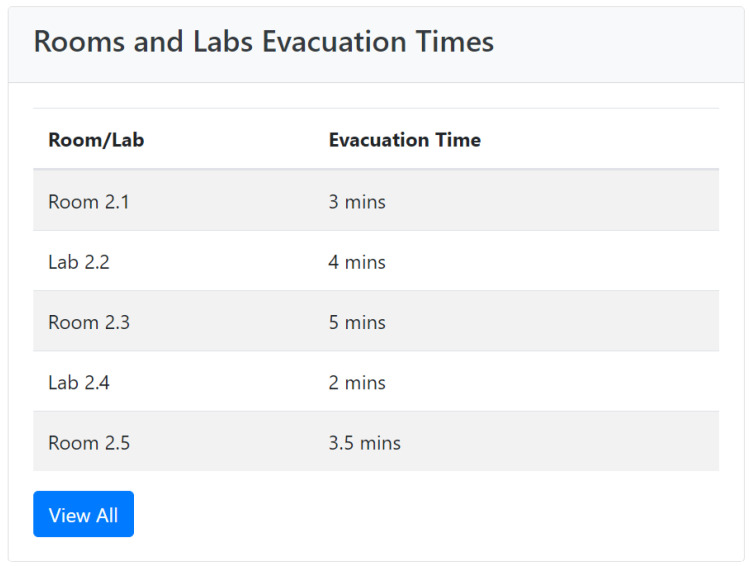
Emergency Analysis—room and lab evacuation times.

The following component is the Rooms and Labs Evacuation Times, shown in Figure 7. It provides a detailed overview of the evacuation efficiency for each individual room and laboratory within the facility. This section is crucial for understanding how different areas of the building respond during emergencies, and can highlight specific zones that might need further attention or improvements in evacuation protocols. The information is presented in a clean, tabular format, making it easy for users to quickly identify and compare evacuation times across various rooms and labs. Each row in the table corresponds to a specific room or laboratory, listing the room/lab name and its respective evacuation time. Initially, just a subset of rooms and laboratories are displayed. By clicking the “View All” button, users can visualize all the data and have the possibility of ordering and filtering them. This structured layout allows for a straightforward comparison, enabling emergency response teams to pinpoint areas with longer evacuation times and investigate the underlying causes. For instance, if certain labs consistently show longer evacuation times, it may prompt a review of their layout and exit accessibility, or the evacuation training provided to personnel in those areas. This targeted analysis is essential for continuous improvement in emergency preparedness.

**Figure 8:**
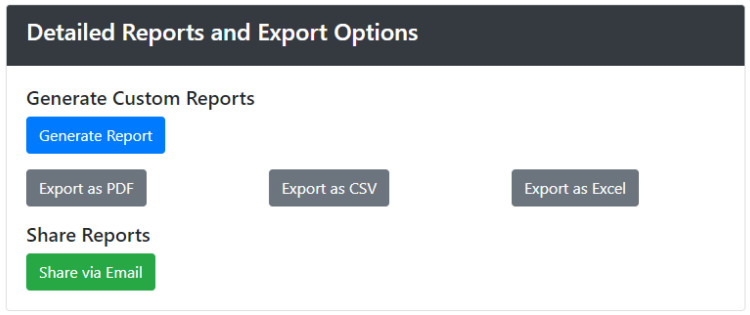
Emergency Analysis—detailed reports.

Finally, the Detailed Reports and Export Options section, depicted in Figure 8, is a powerful feature designed to provide users with comprehensive and customizable reports on emergency data. This section allows users to generate in-depth reports that compile critical information and statistics from various parts of the Emergency Statistics dashboard. These reports are invaluable for post-incident analysis, audits, compliance reviews, and strategic planning, offering a structured and detailed view of emergency management performance. Users can utilize the Report Generation tools to create custom reports based on their specific needs. The interface allows for the selection of various parameters, such as date ranges, incident types, response times, and specific locations within the building or campus. Users can choose which metrics and visualizations to include, such as response efficiency charts, evacuation metrics, and historical trends. This customization ensures that the reports are tailored to meet the unique requirements of different stakeholders, whether they are emergency managers, safety officers, or external auditors. In addition to generating reports, the section provides robust Export Options. Users can export the compiled reports in various formats, including PDF, CSV, and Excel, facilitating easy sharing and further analysis. The PDF format is particularly useful for creating professional and easily distributable documents, while the CSV and Excel formats allow for deeper data manipulation and integration with other analytical tools. The export functionality also includes options to share reports directly via email or cloud services, streamlining the process of disseminating critical information to relevant parties. These export features ensure that all necessary data and insights can be accessed, analyzed, and acted upon efficiently, supporting continuous improvement in emergency preparedness and response.

### 4.3. Usability Evaluation

To evaluate the prototype, we conducted a usability session with eight participants (average age: 29.6±9.1, min: 22, max: 50). Even if the sample size was limited, it is important to remember that a small sample size, typically around five participants, is often sufficient to uncover the majority of usability issues, according to the human–computer interaction literature based on the works of Nielsen [42]. By conducting our initial testing with eight participants, we aimed to achieve remarkable efficiency in identifying and addressing the vast majority of existing problems within the user interface. The session aimed to assess the User Interface usability using the System Usability Scale (SUS) [43]. The SUS is a widely recognized tool for evaluating the usability of a system. It consists of a 10-item questionnaire with five response options for respondents, ranging from “Strongly agree” to “Strongly disagree”. Participation was voluntary, and all participants were informed about their rights regarding participation and data privacy in accordance with the European General Data Protection Regulation. We recruited participants through snowball sampling, and although we could only enlist eight users, the scientific literature suggests that six users can uncover approximately 90% of usability issues under specific conditions [43].

During the session, participants were asked to interact with two new UIs—Floor Map and Emergency Analysis—and to complete predefined tasks, such as determining whether a laboratory was empty or not. After completing these tasks, participants filled out an online questionnaire consisting of the ten SUS items, rated on a 5-point Likert scale (0 = strongly disagree to 4 = strongly agree). The overall SUS score was 89 out of 100, which is significantly above the average benchmark score of 68, indicating high usability.

**Figure 9:**
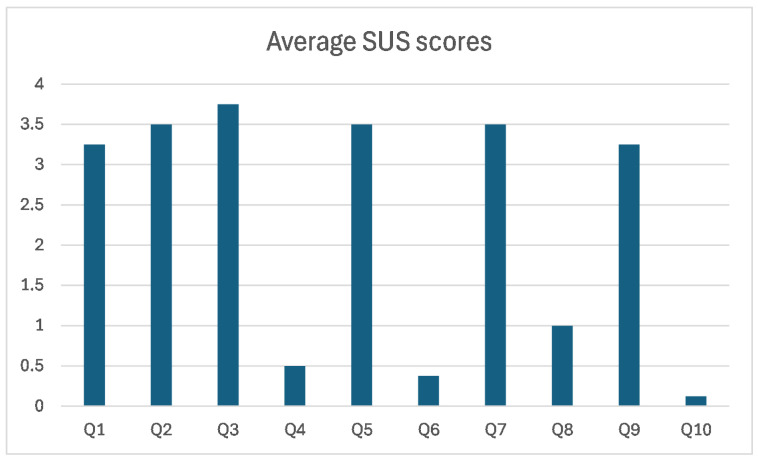
Average SUS scores obtained during the usability evaluation session.

The average score for each SUS question is illustrated in Figure 9. Notably, questions “I thought the system was easy to use” (item #3 in SUS) and “I needed to learn a lot of things before I could get going with the system” (item #10 in SUS) received exceptionally high scores of, respectively, 3.75 out of 4 and 0.125 out of 4. This suggests that participants found the system easy to use, well-integrated, and effective for monitoring building evacuations.

## 5. Conclusions and Future Works

This paper addressed the challenges of emergency management in smart campuses by proposing an improved people-counting system architecture, building upon the scenarios outlined in [25]. Our focus was on developing a tool that could effectively support people during emergencies. To achieve this, we designed and integrated new user interfaces (UIs) specifically tailored for emergency management tasks within the existing web application. A usability evaluation was carried out to validate them.

There are plenty of directions for future works. More tests could be conducted, involving users that have particular responsibilities on the campus during emergencies to gain direct feedback from them. With regard to web applications, data visualization tailored to show the evacuation of people from buildings, showing the flows of people, could be designed and implemented. Moreover, we plan to conduct experimental tests during upcoming emergency evacuation drills on campus to gather real-world data and further validate the system’s effectiveness in managing emergency situations.

## Data Availability

No new data were created or analyzed in this study. Data sharing is not applicable to this article.

## Abbreviations

The following abbreviations are used in this manuscript:

UI	User Interface
LoRa	Long Range
IoT	Internet of Things

## References

[1] Damaševičius R., Bacanin N., Misra S. (2023). From sensors to safety: Internet of Emergency Services (IoES) for emergency response and disaster management. J. Sens. Actuator Netw..

[2] Bujari A., Luglio M., Palazzi C.E., Quadrini M., Roseti C., Zampognaro F. (2020). A virtual PEP for web optimization over a satellite-terrestrial backhaul. IEEE Commun. Mag..

[3] Bandyopadhyay D., Sen J. (2011). Internet of things: Applications and challenges in technology and standardization. Wirel. Pers. Commun..

[4] Masciadri A., Lin C., Comai S., Salice F. (2022). A Multi-Resident Number Estimation Method for Smart Homes. Sensors.

[5] Delnevo G., Roccetti M., Mirri S. (2020). Intelligent and good machines? The role of domain and context codification. Mob. Netw. Appl..

[6] Polin K., Yigitcanlar T., Limb M., Washington T. (2023). The making of smart campus: A review and conceptual framework. Buildings.

[7] Villegas-Ch W., Palacios-Pacheco X., Luján-Mora S. (2019). Application of a smart city model to a traditional university campus with a big data architecture: A sustainable smart campus. Sustainability.

[8] Comai S., Masciadri A., Zuccarello D., Salice F. (2023). NeeMAS: A Need-Based Multi-agent Simulator of Human Behavior for Long-Term Drifts in Smart Environments. Proceedings of the International Conference on Ubiquitous Computing and Ambient Intelligence.

[9] Gupta A., Deokar A., Iyer L., Sharda R., Schrader D. (2018). Big data & analytics for societal impact: Recent research and trends. Inf. Syst. Front..

[10] Sienkiewicz-Małyjurek K., Owczarek T. (2020). Complementarity of communication and coordination in ensuring effectiveness of emergency management networks. Sustainability.

[11] Kamruzzaman M., Sarkar N.I., Gutierrez J., Ray S.K. (2017). A study of IoT-based post-disaster management. Proceedings of the 2017 International Conference on Information Networking (ICOIN).

[12] Hiremath S., Yang G., Mankodiya K. (2014). Wearable Internet of Things: Concept, architectural components and promises for person-centered healthcare. Proceedings of the 2014 4th International Conference on Wireless Mobile Communication and Healthcare-Transforming Healthcare through Innovations in Mobile and Wireless Technologies (MOBIHEALTH).

[13] Motlagh N.H., Bagaa M., Taleb T. (2017). UAV-based IoT platform: A crowd surveillance use case. IEEE Commun. Mag..

[14] Alhaddad M.M. (2019). Improving security performance in smart campuses. Res. Rev. Sci. Technol..

[15] Delnevo G., Mirri S., Prandi C., Manzoni P. (2023). An evaluation methodology to determine the actual limitations of a TinyML-based solution. Internet Things.

[16] Ridwan W.M., Sapitang M., Aziz A., Kushiar K.F., Ahmed A.N., El-Shafie A. (2021). Rainfall forecasting model using machine learning methods: Case study Terengganu, Malaysia. Ain Shams Eng. J..

[17] Sankaranarayanan S., Prabhakar M., Satish S., Jain P., Ramprasad A., Krishnan A. (2020). Flood prediction based on weather parameters using deep learning. J. Water Clim. Chang..

[18] Sreelakshmi S., Chandra S.V. (2022). Machine Learning for Disaster Management: Insights from past research and future implications. Proceedings of the 2022 International Conference on Computing, Communication, Security and Intelligent Systems (IC3SIS).

[19] Ragini J.R., Anand P.R., Bhaskar V. (2018). Big data analytics for disaster response and recovery through sentiment analysis. Int. J. Inf. Manag..

[20] AbuAlnaaj K., Ahmed V., Saboor S. A strategic framework for smart campus. Proceedings of the International Conference on Industrial Engineering and Operations Management.

[21] Alqourabah H., Muneer A., Fati S.M. (2021). A smart fire detection system using IoT technology with automatic water sprinkler. Int. J. Electr. Comput. Eng..

[22] Ali Z., Shah M.A., Almogren A., Ud Din I., Maple C., Khattak H.A. (2020). Named data networking for efficient iot-based disaster management in a smart campus. Sustainability.

[23] Sasirekha V., Ramadevi R., Malarvizhi C., Mohankumar N., Velmurugan S. (2024). Intelligent Campus Safety Management using IoT and CNNs for Surveillance, Access, and Emergency Response. Proceedings of the 2024 3rd International Conference for Innovation in Technology (INOCON).

[24] Gill S.H., Razzaq M.A., Ahmad M., Almansour F.M., Haq I.U., Jhanjhi N., Alam M.Z., Masud M. (2022). Security and privacy aspects of cloud computing: A smart campus case study. Intell. Autom. Soft Comput..

[25] Delnevo G., Mirri S., Salomoni P., Ghini V. (2023). Emergency Management in Smart Campus: Case Studies and Future Directions. Proceedings of the 2023 International Conference on Information and Communication Technologies for Disaster Management (ICT-DM).

[26] Dugdale J., Moghaddam M.T., Muccini H. (2021). Iot4emergency: Internet of things for emergency management. ACM SIGSOFT Softw. Eng. Notes.

[27] Liu Z., Wang C. (2019). Design of traffic emergency response system based on internet of things and data mining in emergencies. IEEE Access.

[28] Cheikhrouhou O., Koubâa A., Zarrad A. (2020). A cloud based disaster management system. J. Sens. Actuator Netw..

[29] Jia D., Wu Z. (2020). Intelligent evaluation system of government emergency management based on BP neural network. IEEE Access.

[30] Yang L., Yang S.H., Plotnick L. (2013). How the internet of things technology enhances emergency response operations. Technol. Forecast. Soc. Chang..

[31] Elvas L.B., Mataloto B.M., Martins A.L., Ferreira J.C. (2021). Disaster management in smart cities. Smart Cities.

[32] Romano M., Díaz P., Aedo I. (2016). Emergency management and smart cities: Civic engagement through gamification. Proceedings of the Information Systems for Crisis Response and Management in Mediterranean Countries: Third International Conference, ISCRAM-med 2016.

[33] De Nicola A., Melchiori M., Villani M.L. (2019). Creative design of emergency management scenarios driven by semantics: An application to smart cities. Inf. Syst..

[34] Ford D.N., Wolf C.M. (2020). Smart cities with digital twin systems for disaster management. J. Manag. Eng..

[35] Zhang Y., Yip C., Lu E., Dong Z.Y. (2022). A Systematic Review on Technologies and Applications in Smart Campus: A Human-Centered Case Study. IEEE Access.

[36] Munawar H.S., Qayyum S., Ullah F., Sepasgozar S. (2020). Big data and its applications in smart real estate and the disaster management life cycle: A systematic analysis. Big Data Cogn. Comput..

[37] Bukar U.A., Jabar M.A., Sidi F. Crisis informatics in smart campus: Opportunities, challenges, and future directions. Proceedings of the International Symposium on ICT Management and Administration (ISICTMA2019).

[38] Ramírez-García J., Ibarra-Orozco R.E., Argüelles Cruz A.J. (2020). Tweets monitoring for real-time emergency events detection in smart campus. Proceedings of the Mexican International Conference on Artificial Intelligence.

[39] Hannan A., Arshad S., Azam M.A., Loo J., Ahmed S.H., Majeed M.F., Shah S.C. (2018). Disaster management system aided by named data network of things: Architecture, design, and analysis. Sensors.

[40] Narendrakumar T., Pillai A.S. (2017). Smart connected campus. Proceedings of the 2017 International Conference on Intelligent Computing, Instrumentation and Control Technologies (ICICICT).

[41] Monti L., Tse R., Tang S.K., Mirri S., Delnevo G., Maniezzo V., Salomoni P. (2022). Edge-Based Transfer Learning for Classroom Occupancy Detection in a Smart Campus Context. Sensors.

[42] Turner C.W., Lewis J.R., Nielsen J. (2006). Determining usability test sample size. Int. Encycl. Ergon. Hum. Factors.

[43] Nielsen J., Landauer T.K. A mathematical model of the finding of usability problems. Proceedings of the INTERACT’93 and CHI’93 Conference on Human Factors in Computing Systems.

